# The added value of ZTE MRI in the assessment of diabetic foot disease

**DOI:** 10.1007/s00256-026-05193-4

**Published:** 2026-03-18

**Authors:** Consolato Gullì, Giuseppe Ferrara, Mario Di Diego, Alessandro Maria Costantini, Emanuele Ferravante, Amato Infante, Daniele Perla, Dario Pitocco, Luigi Natale

**Affiliations:** 1 Advanced Radiology Center, Department of Diagnostic Imaging and Radiation Oncology, Fondazione Policlinico Universitario A. Gemelli IRCCS, L. Go A. Gemelli, 8, 00168 Rome, Italy; 2 Section of Radiology, Department of Radiological and Hematological Sciences, Università Cattolica del Sacro Cuore, Rome, Italy; 3 Diabetes Care Unit, Department of Medical and Surgery Sciences, Fondazione Policlinico Universitario A. Gemelli IRCCS, Rome, Italy

**Keywords:** ZTE, Diabetic foot, Charcot foot, Osteomyelitis, CT

## Abstract

**Objectives:**

To analyze the added value of Zero Echo Time (ZTE) MRI compared with a standard MRI protocol in detecting osseous alterations in diabetic foot. To compare ZTE performance with CT for detecting osseous abnormalities and to evaluate inter-observer reproducibility using Cohen’s kappa coefficient.

**Materials and methods:**

This retrospective study included 46 patients (33 men, 13 women; median age, 64 years) with clinically confirmed diabetes mellitus and clinically suspected diabetic foot complications. A standard MRI protocol including a ZTE sequence was performed in all patients; all patients also underwent CT for comparison. Three readers (two radiologists and one radiology resident) independently assessed image quality and osseous abnormalities, including bone erosions, bone and soft tissue pneumatosis, dislocations, and sclerosis. Sensitivity, specificity, accuracy, and inter-observer agreement (Cohen’s κ) were calculated.

**Results:**

ZTE demonstrated superior detection in almost all osseous alterations compared with the standard MRI protocol, with the exception of bone exposure and erosions, where performance was comparable. Image quality of ZTE was rated as good to excellent in the majority of examinations. Inter-observer agreement for ZTE was excellent between the two musculoskeletal radiologists and ranged from moderate to excellent between the senior radiologist and the resident. ZTE showed high concordance with CT for detection of bone abnormalities.

**Conclusion:**

ZTE significantly improves detection of osseous alterations in diabetic foot, even under suboptimal imaging conditions. Its high image quality and strong inter-reader agreement support integration into standard MRI protocols for improved bone assessment.

## Introduction

Among the various complications associated with diabetes, musculoskeletal disorders, particularly those involving the feet, are a significant cause of morbidity and disability [1, 2]. Neuropathic arthropathy, also known as Charcot foot, is one of the primary musculoskeletal manifestations of diabetes, affecting 0.15%—2.5% of all individuals [3–7], and it is primarily seen in patients with long-standing diabetes who have developed peripheral neuropathy and vascular disease [5, 6]. It is characterized by progressive destruction of the bones and joints in the foot and ankle, often resulting from repetitive trauma and stress that is undetected due to the loss of protective sensation in the affected limbs, which can eventually result in the collapse of the foot’s structural integrity [6]. Another major complication associated with diabetes is the increased risk of foot ulceration, with the lifetime risk of developing a foot ulcer being as high as 34% [8]. Ulcers can serve as entry points for infections, which, if left untreated, can progress to more severe complications such as osteomyelitis [8, 9], which represents a grave clinical concern, as it is associated with a high risk of amputation [10]. It is not uncommon for Charcot foot and osteomyelitis to coexist, creating diagnostic challenges due to the overlap in clinical and radiological findings [9, 10].

The clinical diagnosis of diabetic foot complications is challenging, since many of these manifestations could share the same clinical presentation, and relies heavily on imaging studies [10]. MRI techniques in a standard diagnostic MRI protocol (T1 and T2-weighted images and STIR sequences), while valuable, could have limitations in evaluating cortical bone and its associated alterations, particularly in advanced-stage cases where severe bone destruction significantly limits the capability of standard T1-weighted images to provide adequate morphological evaluation of residual osseous structures [8].

Recent advances have led to the development of novel sequences, such as Zero Echo Time (ZTE), which ensures immediate data acquisition after the radiofrequency pulse, allowing for the visualization of structures with very short T2 times, such as osseous structures and calcifications [11–14]. One of the fundamental advantages of ZTE is its ability to generate isotropic, multiplanar images, similar to CT, and 3D volume rendering, offering enhanced visualization of complex osseous structures [11, 12, 14, 15].

Despite these advantages, ZTE sequences have some limitations due to artifacts, particularly from gas. Gas in the joints or tissues appears as a signal-free region in the images, which can be erroneously interpreted as calcifications or ossifications during the gray-scale inversion process [11].

## Materials and methods

### Study protocol and patient population

The Local Ethics Committee approved this retrospective observational single-centre study (no. 27023–2).

A retrospective study was conducted analysing MRI studies performed between March 2024 and March 2025. The inclusion criteria for this study were age > 18 years, a confirmed clinical and laboratory diagnosis of diabetes mellitus and the presence of a CT of the same foot, which was used for comparative analysis. The exclusion criteria were the patient's inability to undergo MRI (non-compatible devices, claustrophobia). The median time interval between CT and MRI was 7 days (IQR: 5.5–18.5 days; range: 3–40 days).

#### MRI imaging protocol

MRI exams were performed on 1.5 T scanners from the same vendor (GE Healthcare, Chicago, IL) with the foot coil for excitation and signal reception. All MRI exams included: sagittal T1-WI fast spin echo (FSE), covering the entire foot; sagittal short tau inversion recovery (STIR) covering the entire foot; 3) coronal STIR and axial T1-WI FSE either on the midfoot and hindfoot or midfoot and forefoot, based on the clinical suspicion; axial ZTE on the entire foot. Intravenous gadolinium was administered in cases of suspected osteomyelitis. (Gadoteridol, 0.2 ml/kg). Detailed acquisition protocol is reported in Table 1.

**Table 1:** Detailed MRI acquisition protocol for diabetic foot pathology

PARAMETER	Sagittal T1-W	Sagittal T2 STIR	Coronal T2 STIR	Axial T1-W	Axial ZTE	Axial T1 FS-W C +	Coronal T1 FS-W C +	Axial T2-W*
Sequence	FSE-XL	FSE-XL	FSE-XL	FSE-XL	SPGR	FSE-XL	FSE-XL	FSE-XL
Echo time (msec)	Minimum Full	50	50	Minimum Full	≈ 0	Minimum Full	Minimum Full	102
Repetition time (msec)	584	5821	4743	611	/	657	634	6634
Inversion time (msec)	/	140	140	/	/	/	/	/
Flip angle	/	/	/	/	2	/	/	/
No. of sections	37	37	57	36	/	36	57	36
Receiver bandwidth (kHz)	31.25	25	25	27.78	62.50	27.78	27.78	62.50
Echo train length	5	15	15	5	/	5	6	15
Imaging range								
Field of view (FOV)	27	27	27	20	27	20	20	20
Section thickness (mm)	3	3	3	3	1.25	3	3	3
Section spacing (mm)	0.3	0.3	0.3	0.3	0	0.3	0.3	0.3
Matrix size	384 × 384	312 × 260	312 × 260	400 × 400	256 × 256	384 × 384	384 × 384	416 × 352
Phase direction	R/L	R/L	A/P	S/I	S/I	S/I	S/I	S/I
Acquisition time (min:sec)	2:40 min	4:51 min	4:45 min	2:35 min	3:37 min	3:31 min	5:59 min	3:44 min

*FSE* Fast spin echo, *STIR* Short tau inversion recovery, *FS* Fat saturation, + *C* with Gadolinium

*Axial T2-W sequence was acquired when the clinical suspicion involved the hindfoot

#### CT imaging protocol

CT examinations were performed with patients in the supine position. A scout view of the whole foot was acquired, and scan coverage extended from the tip to the posterior margin of the calcaneal skin. The scan was conducted in a craniocaudal direction, with a minimum tube voltage of 120 kVp and a tube current of 100 mAs. Field of view (FOV) ranged from 100 to 160 mm and was adjusted when necessary to improve in-plane resolution. Axial acquisitions were obtained with a slice thickness of 0.625 mm and an interslice interval of 0.3 mm, using bone and soft tissue reconstruction algorithms.

### Image analysis

MRI and CT scans were retrieved from our Picture Archiving and Communication System (PACS) (Carestream Vue PACS, Philips, USA).

All MRI and CT examinations were reviewed independently and blindly by three readers: two radiologists sub-specialised in musculoskeletal imaging (R1, with 30 years of experience; R2, with 5 years of experience) and one radiology resident with a specific interest in MSK radiology (R3).

The readers assessed the presence of specific bone abnormalities relevant to diabetic foot disease: soft tissue pneumatosis, bone pneumatosis, bone erosions, bone exposure, joint dislocations, sclerosis, periosteal reaction, and bone fragmentation, that have been defined in Table 2.

**Table 2:** MRI-based definitions of principal bone and peri-bone abnormalities in the diabetic foot

Abnormality	MRI-Specific Definition
Soft tissue and bone pneumatosis	Focal signal voids (dark areas) on all pulse sequences, often with blooming artifact on susceptibility-sensitive sequences. This finding suggests gas-forming infection (e.g. necrotizing infection or wet gangrene) in the soft tissue or in the bone (emphysematous osteomyelitis) [8, 16, 17].
Bone erosions	Focal loss of cortical or subchondral bone, usually at joint margins. Erosions are seen as a breach in the normal bone cortex or subchondral bone plate with adjacent marrow signal abnormality (T2 hyperintensity with variable T1 signal) at the erosion margins [16, 18].
Bone exposure	Direct exposure of bone to the external environment via an ulcer. On MRI this is indicated by an ulcer extending continuously from the skin surface to the underlying bone cortex [16].
Joint dislocations	Complete loss of normal alignment between articular surfaces. MRI depicts dislocation as gross malalignment of the joint—with bones displaced from their usual positions. Dislocations on MRI often coexist with fractures and collapse in advanced neuropathic arthropathy [19].
Sclerosis	Abnormal bone hardening and increased density, which contains little mobile water and thus appears dark on MRI. Sclerotic bone is characteristically low in signal intensity on both T1- and T2-weighted images (“eburnation” of bone) [18].
Periosteal reaction	Reactive new bone formation along the periosteum in response to infection or trauma. On MRI, periosteal reaction can manifest as a linear or lamellar elevation of the periosteum with adjacent signal changes; however, it is more easily recognized on radiographs/CT and in ZTE sequences [18].
Bone fragmentation	Severe osteodestructive change wherein bone is broken into multiple fragments. On MRI this corresponds to the acute fragmentation phase of Charcot neuro-osteoarthropathy: there are multiple separated bone pieces, cortical fractures, and collapse, often with surrounding oedema [19–21].

The image quality of the ZTE sequences was assessed using a 5-point Likert scale. Each examination was rated according to the following criteria: "Very Good" (score 4), indicating optimal depiction of all structures without any image degradation; "Good" (score 3), denoting minimal image deterioration with preservation of all structural details; "Adequate" (score 2), representing overall acceptable image quality with minor areas of compromise; "Poor" (score 1), defined as partial obscuration of anatomical structures; and "Very Poor" (score 0), indicating complete obscuration of the imaged structures.

### Clinical data

The following clinical information were retrieved from hospital records: diabetes status, blood pressure (BP), age. Diabetes was diagnosed in case of the following laboratory test: A1C of greater than or equal to 6.5%, fasting blood glucose of greater than or equal to 126 mg/dl and two-hour blood glucose of greater than or equal to 200 mg/dl. Hypertension was diagnosed for systolic BP values ≥ 140 mmHg and/or diastolic BP values ≥ 90 mmHg.

### Statistical analysis

Data analysis was performed using IBM SPSS Statistics version 24. Descriptive statistics were reported as absolute values, percentages, and relative frequencies, including subgroup analyses were deemed appropriate. Diagnostic performance of the parameters under investigation was assessed through the calculation of sensitivity, specificity, positive predictive value (PPV), negative predictive value (NPV), and overall diagnostic accuracy. Inter-observer agreement was evaluated using Cohen’s kappa (κ) coefficient. Results were graphically represented using simple two-way tables and polar charts to enhance interpretability and comparative visualization.

## Results

### Patient characteristics

**Fig. 1:**
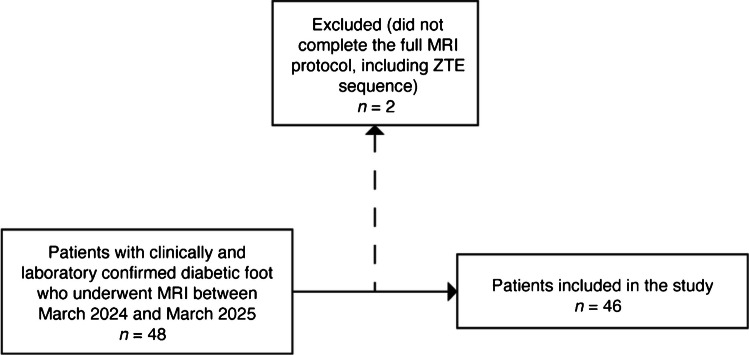
Flowchart illustrating patient selection for the study

A total of 48 patients were screened and met the inclusion criteria for the present study. Two patients were excluded due to prematurely interrupting the exam before the ZTE sequence was performed. A total of 46 patients were retrospectively included in the study; the population is composed of 33 males and 13 females, with a median age of 64 years (Fig. 1).

Among the included patients, arterial hypertension was present in 38 out of 46 individuals (83%). 43 patients (93%) had type 2 diabetes mellitus, while only 3 patients (7%) had type 1 diabetes. The patient cohort was stratified into four subgroups based on the patient’s diagnosis: patients with Charcot foot without osteomyelitis (*n* = 12), patients with osteomyelitis without Charcot foot (*n* = 11), patients with both osteomyelitis and Charcot neuroarthropathy (*n* = 10) and patients without either condition but presenting with other diabetic foot–related complications (*n* = 13).

#### Imaging findings and comparison with a standard MRI protocol

**Fig. 2:**
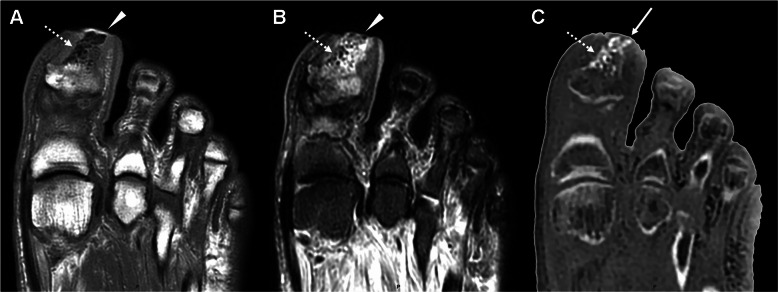
Forefoot ulcer with bone erosions and pneumatosis in a 71-year-old male. Axial T1 (**A**), axial STIR (**B**), and axial ZTE (**C**) images demonstrate an ulcer of the hallux (arrowhead in A and B), with cortical erosion of the distal phalanx (arrow in C), and intramedullary pneumatosis in the distal half (dashed arrow in A, B and C)

**Fig. 3:**
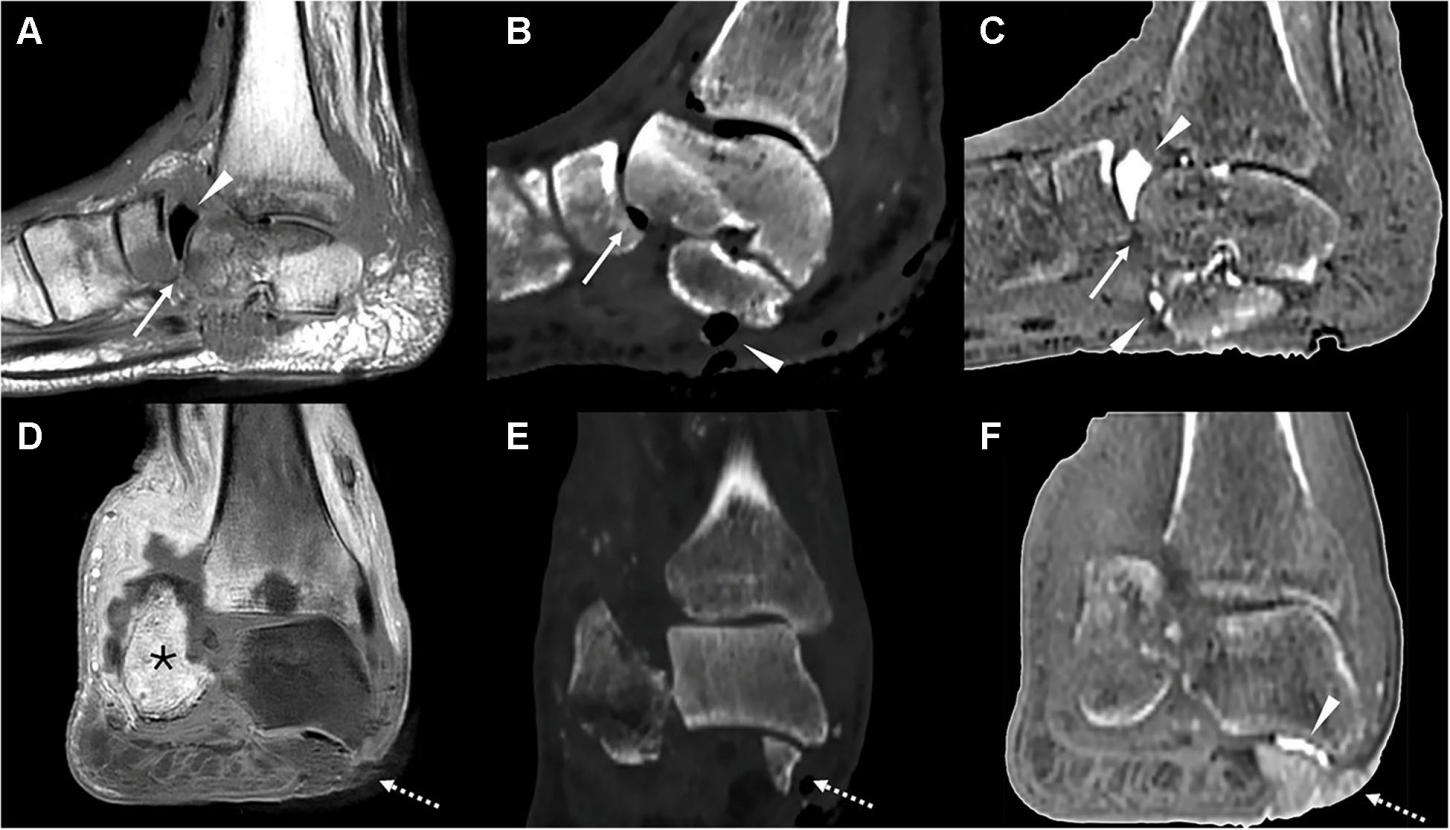
Late-stage Charcot foot with superimposed osteomyelitis in a 74-year-old male. Sagittal T1 (**A**), sagittal CT multiplanar reconstruction (**B**), sagittal ZTE reconstruction (**C**), coronal T1 post-contrast (**D**), coronal CT multiplanar reconstruction (**E**), and coronal ZTE reconstruction (**F**) images demonstrate severe hindfoot destruction with calcaneal fragmentation and erosion, talonavicular subluxation (arrow in A, B and C) and subtalar dislocation, soft tissue pneumatosis (arrowheads in A, B, C and F), and a hindfoot ulcer with exposure of the talus (dashed arrow in D, E and F). Note the diffuse bone contrast enhancement, particularly involving the calcaneus, indicative of osteomyelitis (*), as well as the enhancement of adjacent soft tissues, consistent with cellulitis

**Fig. 4:**
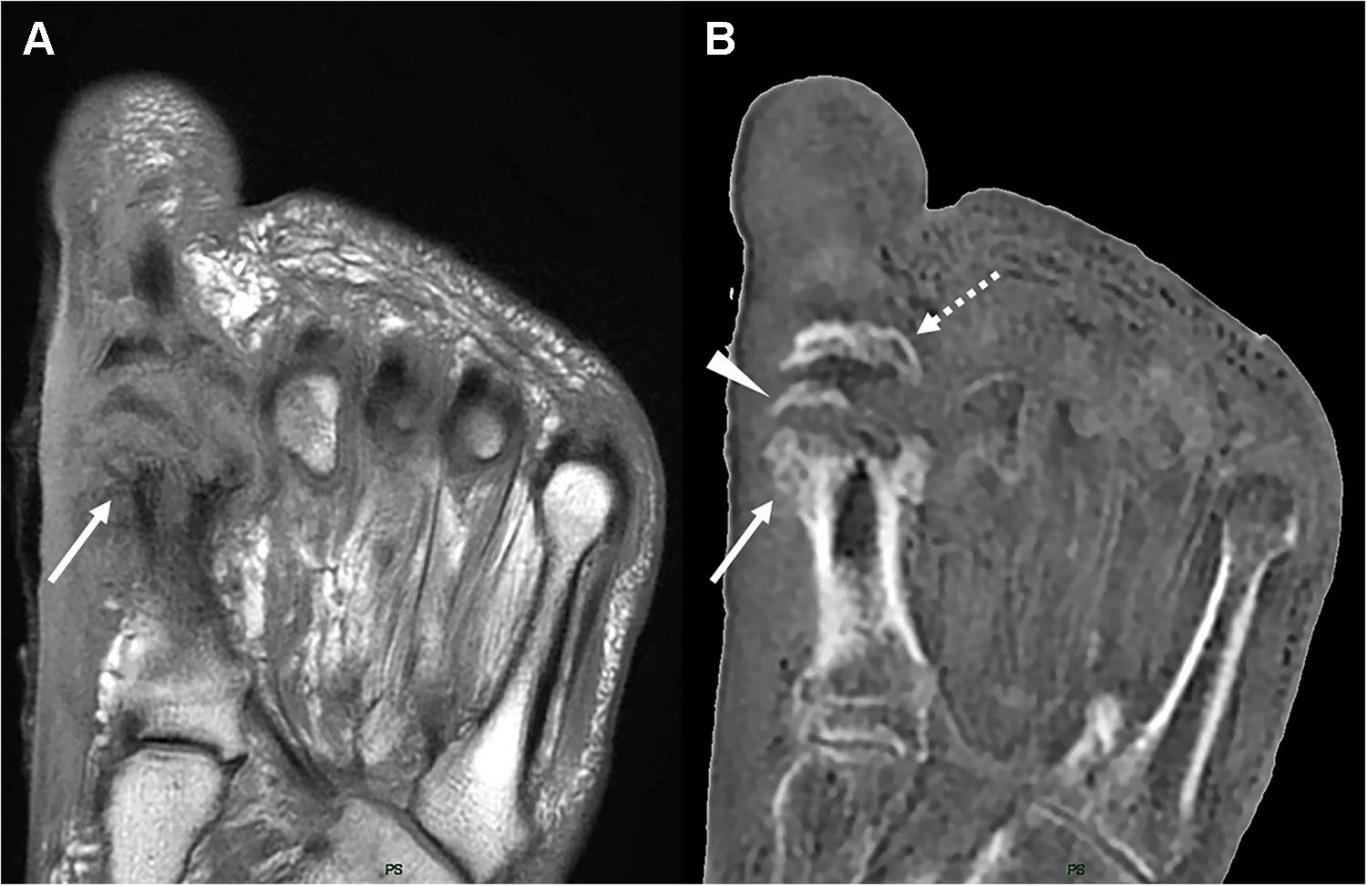
Forefoot osteomyelitis with bone erosions in a 49-year-old male. Axial T1 (**A**) and axial ZTE (**B**) images show severe destruction of the first metatarsophalangeal joint with extensive erosions of the metatarsal head (arrow in A and B) and deformity of the proximal phalanx (dashed arrow in B), as well as a bone fragment dislocated in the articular space (arrowhead in B)

**Fig. 5:**
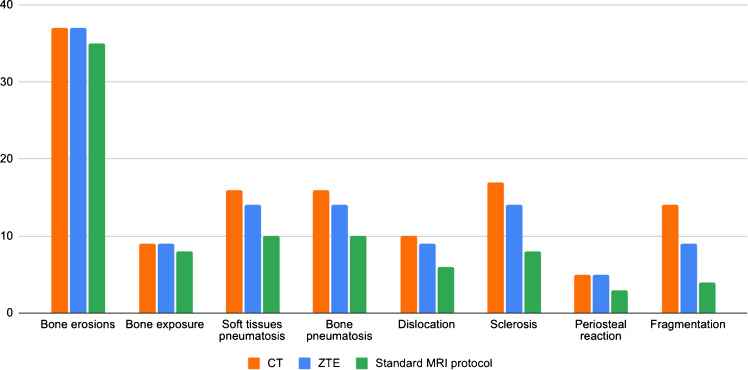
Bar chart showing the number of cases in which each osseous abnormality was detected by CT, ZTE, and a standard MRI across the study cohort

Overall MRI findings demonstrated prevalent osseous abnormalities, with notable occurrences including bone erosions (37 cases), sclerosis (14 cases), joint dislocations (9 cases), soft tissue pneumatosis (14 cases), bone pneumatosis (14 cases), bone exposures (9 cases), periosteal reactions (5 cases), and bone fragmentation (9 cases). ZTE sequences demonstrated superior efficacy in delineating specific abnormalities compared to a standard MRI protocol. Notably enhanced detection was observed for soft tissue pneumatosis (14 cases vs. 10), bone pneumatosis (14 cases vs. 10), joint dislocations (9 cases vs. 6), sclerosis (14 cases vs. 8), periosteal reactions (5 cases vs. 3), and bone fragmentation (9 cases vs. 4) (Figs. 2, 3,  4). A comprehensive overview of case numbers is provided in Fig. 5.

#### ZTE image quality

Overall, ZTE image quality was rated as high in most cases, with R1 assigning the maximum Likert score of 4 in 31 exams (67.4% of the cohort). Scores of 3 were reported in 7 exams (15.2%), while lower ratings were less common, with 6 exams (13.0%) rated as 2 and only 2 exams (4.3%) rated as 1. No examinations were assigned a score of 0.

#### Subgroup analysis of cases with low ZTE image quality

To further investigate the diagnostic robustness of ZTE sequences, a dedicated subgroup analysis was performed on examinations rated as low quality (Likert score ≤ 2). Within this cohort, we specifically identified cases in which ZTE sequences enabled the detection of osseous abnormalities that were not appreciated on standard protocol MRIs by any of the three readers. Despite the technical limitations inherent to suboptimal ZTE image quality, this sequence still facilitated the identification of additional bone alterations, most notably bone erosions (*n* = 4), bone pneumatosis (*n* = 2), bone fragmentation (*n* = 2), and sclerosis (*n* = 1) (Table 3).

**Table 3:** Additional osseous abnormalities identified exclusively by ZTE (not detected on standard protocol sequences) in examinations with low ZTE image quality (Likert ≤ 2)

Findings	Cases detected only by ZTE with low image quality
Soft tissue pneumatosis	0
Bone pneumatosis	2
Bone erosions	4
Bone exposure	0
Joint dislocations	0
Sclerosis	1
Periosteal reaction	0
Bone fragmentation	2

#### Imaging findings: ZTE vs. CT

In the analysis comparing ZTE MRI findings against CT as the reference standard, ZTE showed perfect agreement with CT (100% sensitivity and specificity) for the detection of bone erosions, bone exposure, and periosteal reaction. Detailed diagnostic performance metrics including positive predictive value (PPV), negative predictive value (NPV), **and Overall Diagnostic Accuracy (ODA)** are presented in Table 4.

**Table 4:** Diagnostic performance of ZTE MRI for detecting bone abnormalities using CT as reference standard

	Soft tissues pneumatosis	Bone pneumatosis	Erosions	Exposure	Dislocations	Sclerosis	Periosteal reaction	Fragmentation
*SENS*	86%	82%	100%	100%	83%	78%	100%	64%
*SPEC*	100%	100%	100%	100%	95%	94%	100%	100%
*PPV*	100%	100%	100%	100%	94%	95%	100%	100%
*NPV*	86%	88%	100%	100%	87%	75%	100%	61%
*ODA*	92%	92%	100%	100%	90%	85%	100%	77%

Metrics shown include SENS (Sensitivity), *SPEC* (Specificity), *PPV* (Positive Predictive Value), *NPV* (Negative Predictive Value), and *ODA* (Overall Diagnostic Accuracy)

### Interobserver agreement

**Fig. 6:**
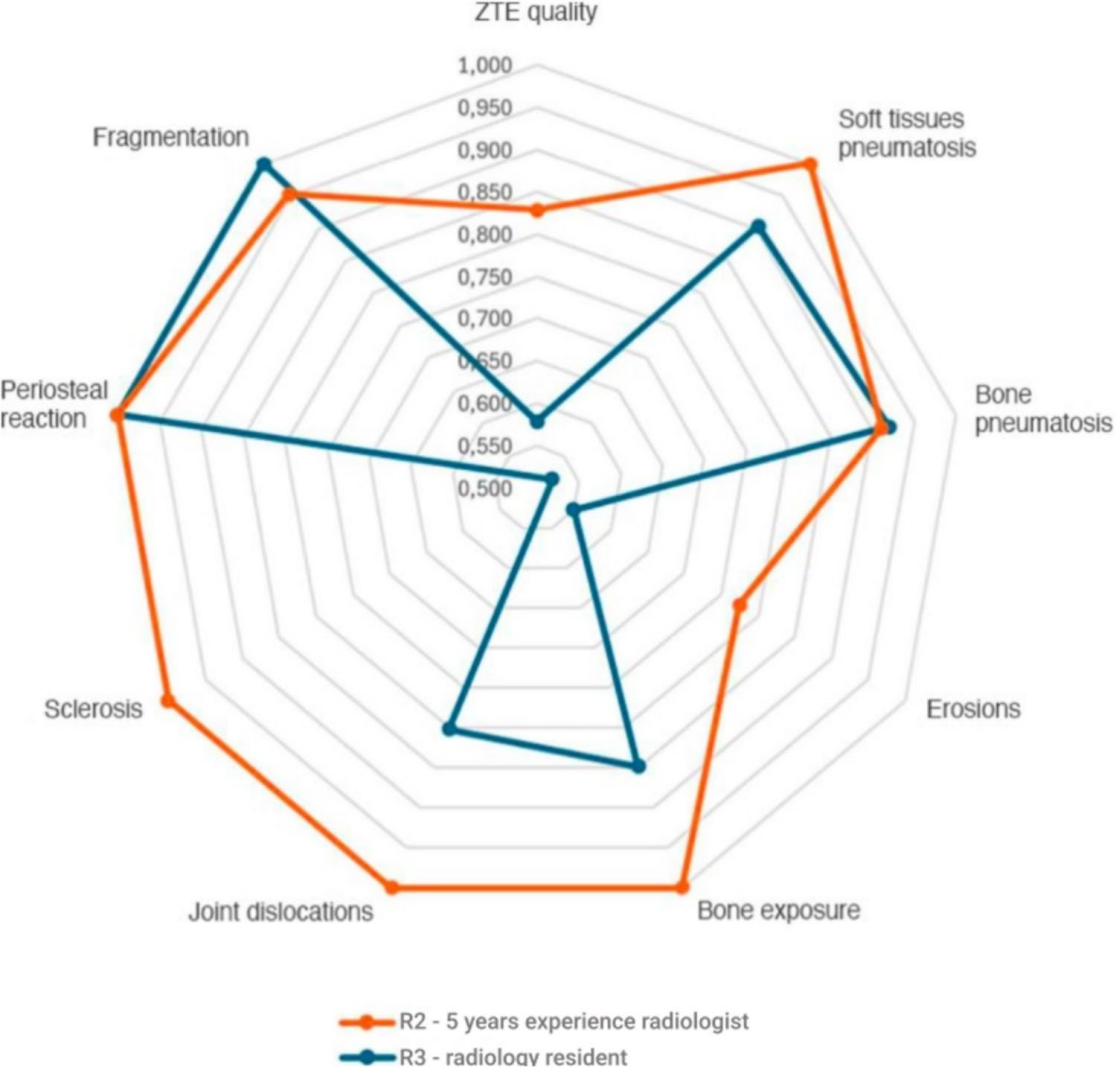
Radar chart illustrating interobserver agreement (Cohen's Kappa) for Detection of Bone Abnormalities on ZTE Sequences. The chart compares the agreement levels of Reader 2 (orange line; 5 years experience) and Reader 3 (blue line; radiology resident) relative to Reader 1 (R1, 30 years experience) across the different assessed findings. Values plotted further from the centre indicate higher agreement, with the outermost edge representing perfect agreement (Kappa = 1.0)

Interobserver agreement for the detection of bone abnormalities on ZTE sequences was assessed using Cohen's kappa (K) statistic, comparing the readings of R2 and R3 against R1 as the reference. The agreement between the two musculoskeletal radiologists (R1 and R2) was excellent for most findings, with K values ranging from 0.775 to 1.000. The agreement between the senior radiologist (R1) and the resident (R3) ranged from moderate to excellent, with K values ranging from 0.479 to 1.000. Detailed interobserver agreement results are shown in Fig. 6.

## Discussion

Although ZTE sequences have been increasingly applied in musculoskeletal imaging, their systematic use in the evaluation of diabetic foot complications has not been previously investigated. This study demonstrates that the implementation of a ZTE MRI sequence significantly enhances the detection of clinically relevant osseous alterations in diabetic foot-related disease, with high diagnostic performance even under variable image quality conditions.

Our results are consistent with previous literature that has established the superior performance of ZTE for cortical bone imaging and the detection of subtle osseous pathology compared to conventional MRI sequences [11–13]. The ability to reconstruct isotropic, high-resolution images in multiple planes was particularly beneficial for the evaluation of joint dislocations, a key complication in Charcot neuro-osteoarthropathy.

In the evaluation of pneumatosis, the typical ZTE artifact of air hyperintensity proved advantageous, allowing better visualization of sinus tracts extending from skin ulcers, showing how technical features generally considered artifacts may facilitate the detection of clinically relevant findings.

The Likert analysis of image quality revealed that most ZTE examinations achieved high scores, indicating excellent depiction of anatomical structures. Even in the minority of cases rated as low quality (Likert ≤ 2), ZTE sequence remained diagnostically valuable, detecting additional bone alterations—such as erosions, bone pneumatosis, and fragmentation—not visible on standard protocol MRI images.

Comparison with CT as a reference standard for the detection of osseous alterations [8, 10, 20–22] confirmed the high sensitivity and specificity of ZTE for detecting bone erosions, bone exposure, and periosteal reaction, with diagnostic accuracy reaching 100% for these findings in our cohort. Performance for bone fragmentation and sclerosis was slightly lower, likely reflecting the challenges inherent to detecting minute fragments or chronic sclerotic changes, which could also be related to the thinner slice thickness that was available for CT (0.625 mm vs. 1.25 mm for ZTE).

ZTE’s short acquisition times are especially relevant in diabetic patients, who are often elderly, may have limited mobility and discomfort repositioning for an additional CT scan.

Furthermore, avoiding ionizing radiation exposure may benefit patients undergoing serial follow-up examinations, as repeated CT scans could otherwise result in a clinically significant cumulative radiation exposure over time [23–25].

ZTE also demonstrated excellent interobserver agreement, with Cohen’s kappa values indicating substantial to almost perfect concordance among expert readers for all key alterations. Moreover, moderate to excellent concordance was found between the expert radiologist and the radiology resident. This high reproducibility, observed across readers with varying levels of experience, supports the robustness of ZTE assessment in diverse radiological settings.

The study has several limitations, including its retrospective design, single-centre setting, and relatively small sample size. Furthermore, the absence of systematic histological confirmation precludes absolute diagnostic certainty, given it was not routinely performed in our cohort due to its invasive nature and limited feasibility in clinical practice. Instead, diagnoses were established through a composite reference standard integrating MRI findings, clinical assessment, laboratory data, and follow-up, mirroring real-life diagnostic pathways but introducing a degree of uncertainty. Although ZTE is conceptually vendor-agnostic, its clinical implementations are currently proprietary and may require dedicated software packages and compatible system configurations. Equivalent short-TE or zero-TE sequences are available on most major MRI platforms under vendor-specific names; however, acquisition parameters and reconstruction algorithms may differ. This inter-vendor variability may influence reproducibility and represents a potential limitation. Future prospective studies with larger, multicentre cohorts and standardized reference standards, ideally including histopathology, are warranted to confirm and expand upon these findings. Moreover, future developments may include the integration of artificial intelligence tools to optimize ZTE acquisition, potentially reducing scan times and further enhancing image quality and diagnostic performance.

In summary, ZTE MRI significantly improves the detection of osseous alterations in diabetic foot, even under suboptimal imaging conditions. Its consistently high image quality, ability to reveal additional findings, and strong inter-reader concordance support its integration into standard MRI protocols for bone assessment.

## Data Availability

Data sharing is not applicable to this article as no datasets were generated or analyzed during the current study.
